# Changes in Smartphone Usage among Adolescents and Associated Subjective Health Concerns: A Secondary Analysis of the Korea Youth Risk Behavior Survey

**DOI:** 10.3390/children11080890

**Published:** 2024-07-25

**Authors:** Geun Woo Lee, Jongwon Moon, Donghun Lee

**Affiliations:** 1 Department of Ophthalmology, Catholic University of Daegu School of Medicine, Daegu, Republic of Korea; lkw0925@naver.com; 2 Department of Ophthalmology, Daegu Fatima Hospital, Daegu, Republic of Korea; anantos@naver.com

**Keywords:** adolescent, smartphone, health concern

## Abstract

Background: We evaluated changes in the smartphone use rate and time among Korean adolescents and their awareness of associated health problems. Methods: This study was a secondary analysis of the Korea Youth Risk Behavior Survey (2020–2023) conducted by the Korean Disease Control and Prevention Agency. The total number of enrolled adolescents aged 12–18 years was 214,526. Results: The weekly smartphone usage rate increased from 96.4% to 97.1% (*p* = 0.03), with no significant changes observed in weekend usage. The average smartphone use time was 4.7 h on weekdays (*p* = 0.17) and 6.6 h on weekends (*p* = 0.37). Middle school adolescents had a higher weekday use rate than high school adolescents, but the average smartphone use time was significantly less. By 2023, the proportion of adolescents with overdependence was 28% (*n* = 14,672). Additionally, 11.8% (*n* = 6255) responded that they had experienced health problems due to smartphone use. Conclusions: The longer they used their smartphones for on the weekends, the more likely they considered their health to be worse. In conclusion, our youth population needs to be educated on the proper use of smartphones.

## 1. Introduction

Smartphone use can have a pronounced impact during adolescence [1,2,3,4]. Previous studies have shown that excessive smartphone use negatively affects adolescents’ mental health, leading to issues such as reduced self-control [1] and sleep disturbances [4] due to decreased outdoor activities. Moreover, the ability to cope with emergencies and psychological adjustments is crucial for adolescents’ mental well-being [5]. Concerning physical health, long-term smartphone use may lead to a significant increase in thoracic kyphosis and trunk inclination [6] and accelerate changes in the refractive error during growth owing to an increase in the near work time [7].

At ophthalmology clinics, we found that the guardians of adolescent patients often believe their children spend a substantial amount of time on their smartphones and they enquire about the average smartphone use time; therefore, we sought to quantify smartphone use for Korean adolescents and their guardians. Additionally, authors have hypothesized that smartphone usage among adolescents may have changed over time as the smartphones usage rate in Korea is high.

Since 2005, the Korea Disease Control and Prevention Agency has been conducting the Youth Health Behavior Survey to understand the health status of Korean youth and to calculate health indicators; the results have been disclosed to the public. This large-scale survey targets > 50,000 adolescents annually. It included questions related to smartphone use from 2020 to 2023, which are valuable for understanding the health behaviors of adolescents.

Adolescence is a developmental period characterized by multilevel changes and poor self-control associated with risk-taking behaviors [1]. It is important to determine whether adolescents have insight into the impact of smartphone use on their health, particularly eye conditions. Therefore, we analyzed trends in the smartphone use time and subjectively perceived health among Korean adolescents using big data analysis.

## 2. Materials and Methods

This retrospective study was approved by the Institutional Review Board of our hospital. Informed consent was waived due to the secondary nature of the analysis, using publicly released data from the Korea Youth Risk Behavior Survey (2020–2023). These raw data were publicly released by the Korean Disease Control and Prevention Agency. This study adhered to the tenets of the Declaration of Helsinki.

**Figure 1:**
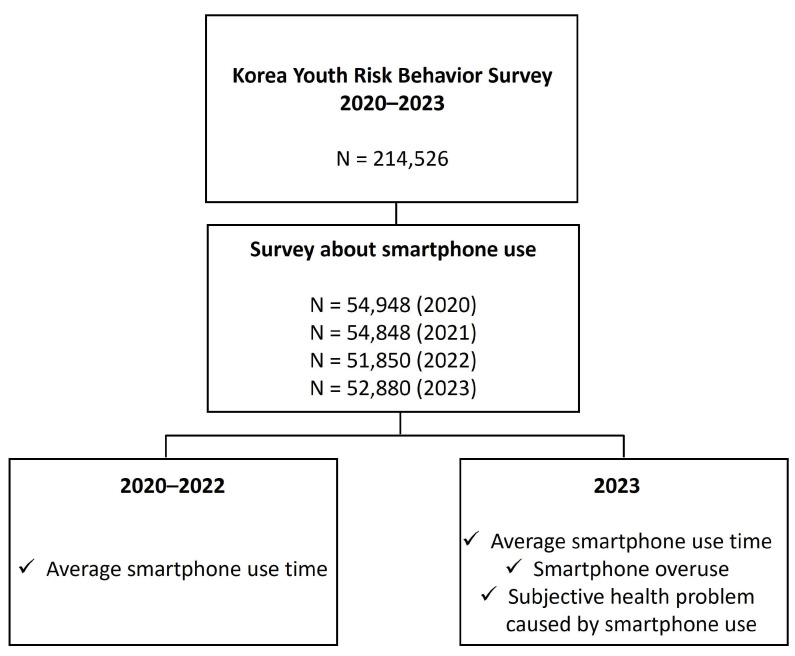
Study population and survey data. A total of 214,526 adolescents participated in the survey from 2020 to 2023. From 2020 to 2022, only the average smartphone use time was surveyed, and in 2023, a survey was conducted on the average smartphone use time, the percentage of excessive use, and the presence of subjective health problems caused by smartphone use.

We secondarily analyzed responses regarding smartphone use from the Korea Youth Risk Behavior Survey conducted by the Korean Disease Control and Prevention Agency, which enrolled Korean adolescents aged 12–18 years. The study included 214,526 Korean adolescents (Figure 1). We examined the smartphone usage rate and average use time on weekdays and weekends over the period from 2020 to 2023. In 2023, questions were added to explore whether smartphone overuse affects health; we analyzed these results as well. The statements in the 10 questions were as follows: (1) you fail every time you attempt to reduce your smartphone use time, (2) it is difficult to control the time spent using your smartphone, (3) it is difficult to adhere to an appropriate smartphone use time, (4) it is difficult to concentrate on other things when your smartphone is beside you, (5) the thought of your smartphone never leaves your head, (6) you feel a strong urge to use your smartphone, (7) you have experienced health problems due to smartphone use, (8) you have experienced severe conflict with your family because of your smartphone use, (9) you have experienced severe conflict with friends and colleagues because of your smartphone use, and (10) you have difficulty working or studying because of your smartphone. Scoring was as follows: 1 point, “not at all”; 2 points, “no”; 3 points, “yes”; and 4 points, “always.” Adolescents who scored >23 out of 40 points were defined as overdependent. The cutoff standard for smartphone overdependence was based on reference presented in the 16th Youth Health Behavior Survey Statistics published by the Korea Disease Control and Prevention Agency. Their proportions were investigated, and those who responded that their health was affected owing to smartphone use were examined.

Statistical analyses were performed using the Statistical Package for Social Sciences (version 25.0; IBM Corp., Armonk, NY, USA). Data comparisons between middle and high school adolescents were performed using the complex-sample chi-square test and a general linear model. To analyze changes in the smartphone use rate and average use time by year, we used a complex-sample general linear model. Complex-sample logistic regression analysis was used to analyze the association between smartphone use and subjective health problems by 2023. Statistical significance was set at *p* < 0.05. The numbers of adolescents are presented as unweighted values, and the percentages in parentheses are presented as weighted values.

## 3. Results

**Figure 2:**
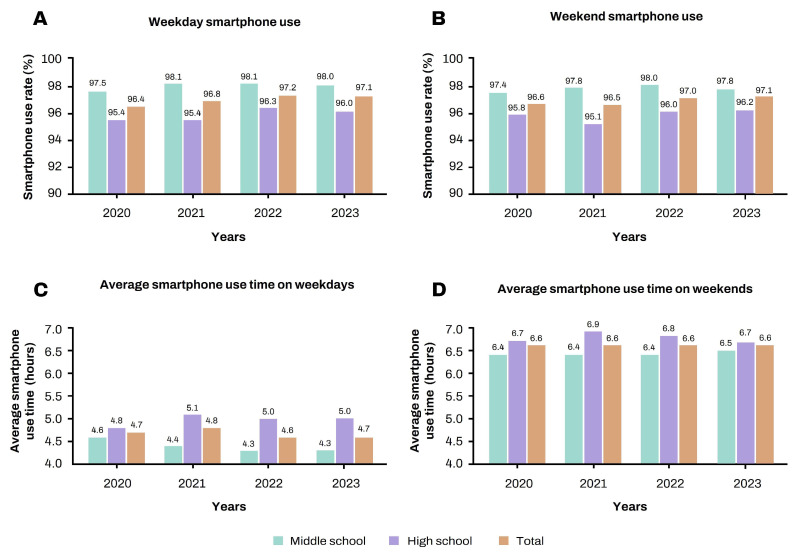
Smartphone use rate and average smartphone use time from 2020 to 2023 among Korean adolescents. (**A**) Weekday smartphone use rate and (**C**) average smartphone use time. (**B**) Weekend smartphone use rate and (**D**) average smartphone use time. The average smartphone use time was significantly higher on weekends than on weekdays every year (*p* < 0.001). On weekdays, no significant change was observed in the smartphone use rate (**A**) and time (**C**) in 2023 versus 2020. Similarly, on weekends, no significant change was observed in the smartphone use rate (**B**) and time (**D**) in 2023 versus 2020.

The smartphone use rate and average use time are shown in Figure 2. The weekday smartphone use rate among Korean adolescents significantly increased from 96.4% in 2020 to 97.1% in 2023 (*p* = 0.03). The weekend use rate increased from 96.6% in 2020 to 97.1% in 2023, although this change was not statistically significant (*p* = 0.06).

The average smartphone use time was 4.7 h on weekdays (*p* = 0.17) and 6.6 h on weekends (*p* = 0.37) in both 2020 and 2023. In all years, middle school adolescents had a higher average weekday smartphone use rate than high school adolescents (97.5–98.1%, middle school; 95.4–96.3%, high school), but the average smartphone use time was significantly less (4.3–4.6 h, middle school; 4.8–5.1 h, high school; *p* < 0.001). On weekends, the average smartphone use rate of middle school adolescents was high (97.4–98.0%, middle school; 95.1–98.0%, high school), but the average smartphone use time was significantly low (6.4–6.5 h, middle school; 6.7–6.9 h, high school; *p* < 0.001).

Table 1 summarizes the smartphone overdependence and subjective health problems by 2023. The proportion of overdependent users was 28% (14,672) of the total number of 52,880 users. Additionally, 11.8% (6255) responded that they had experienced health problems due to smartphone use. No significant difference was observed in the proportion of overdependence between middle and high school adolescents (*p* = 0.545), and the weekday smartphone use time of the overdependent high school adolescents was 5.6 ± 0.1 h, which was significantly longer than the 5.0 h of middle school adolescents (*p* < 0.001).

The associated factors elevating subjective health problems due to smartphone use in 2023 are presented in Table 2. The middle school adolescents had a 1.11 times higher risk of experiencing health problems owing to smartphone use and a higher risk of smartphone overdependence (odds ratio 9.94, *p* < 0.001); a longer smartphone use time on weekends was associated with a higher risk of reporting subjective health problems (odds ratio 1.04, *p* < 0.001).

## 4. Discussion

Overall, South Korea has a high smartphone use rate of 89.5%, with 97.2% of adolescents using smartphones in 2018 [4,8]. This study aimed to evaluate changes in the smartphone usage rates and times among Korean adolescents and their awareness of the health problems associated with smartphone use. Long-term exposure to digital media devices has been linked to various ocular symptoms in children and adolescents, including eye fatigue, blurring, redness, and visual disturbances [9,10]. It also reduces blinking [11] and increases the risk of eye diseases such as esotropia and dry eye syndrome [12,13]. Additionally, radiation from mobile phones can cause oxidative stress in the cornea and lens [14], and LED exposure can decrease the lipid layer thickness in the eyes [15]. Therefore, it is crucial to investigate smartphone use among adolescents, especially as it can affect visual acuity and binocular vision during their developmental phase. Although the Korea Youth Risk Behavior Survey does not include ophthalmic examinations, it provides reliable data due to its accurate sampling methods. Our analysis of Internet use among Korean adolescents from 2009 to 2019 showed an increase from 2.5 h on weekdays and 4.1 h on weekends in 2019 compared to 1.9 h and 3.0 h in 2009, respectively [16]. This follow-up analysis of smartphone use from 2020 to 2023 revealed an average daily use of 4.6 h on weekdays and 6.6 h on weekends in 2023. These values are significantly higher compared to other countries. For example, adolescents in 19 European countries reported an average smartphone use of 167 min in 2020 [17]. In the United States, adolescents with an evening circadian preference used the Internet for 3.8 h on weekdays and 5.4 h on weekends [18]. In 2023, 28% of adolescents were overdependent on smartphones, and 11.8% experienced health problems due to their use. Excessive smartphone use negatively impacts adolescents [1,2,3], underscoring the need for proper education on smartphone use. This is supported by findings that highlight the importance of psychological adjustment and coping mechanisms in maintaining mental health [5]. High school adolescents reported higher average usage times on both weekdays and weekends compared to middle school adolescents, but fewer perceived health impacts. This may indicate a lack of awareness or sensitivity to problematic smartphone use among high school students, warranting increased caution and education.

This study has some limitations. First, our results were based on responses to a survey, and the possibility of a recall bias could not be excluded. Second, because only subjective opinions were analyzed, objective values from ophthalmological examinations, such as refractive errors and visual acuity, could not be analyzed. Third, because information was collected only through the survey, information bias could not be excluded. Fourth, the wording of the survey questions differed slightly depending on the year—this may have affected the analysis. In the 2020–2022 survey, only the time spent on smartphones was surveyed, whereas in 2023, subjective questions regarding dependence on smartphones were included. These differences may have affected our results.

We believe that the results of this study provide meaningful information for clinical care and research because the Youth Health Behavior Survey is a national survey conducted on a large scale with accurate and systematic sampling. 

In conclusion, our analysis shows that the time spent using media devices by Korean adolescents has increased compared with that during the 2010s, and the rate of overdependence is high at 28%. Therefore, it is necessary to provide multifaceted management and support for youth smartphone use.

## Figures and Tables

**Table 1:** Smartphone overdependence and subjective health problems in 2023.

	Total	Middle School	High School	*p* Value
Number of adolescents	52,880	28,401	24,479	
Weekday				
Presence of smartphone use (%)	51,357 (97.1%)	27,839 (98.0%)	23,518 (96.0%)	<0.001 ^1^
Average time of smartphone use (hours)	4.7	4.3	5.0	<0.001 ^2^
Weekend				
Presence of smartphone use (%)	51,341 (97.1%)	27,769 (97.8%)	23,572 (96.2%)	<0.001 ^1^
Average time of smartphone use (hours)	6.6	6.5 ± 0.1	6.7 ± 0.1	<0.001 ^2^
Smartphone overdependence				
Number of adolescents	14,672 (28.0)	7840 (27.8)	6832 (28.2)	0.545 ^1^
Average time of use on weekdays (hours)	5.3	5.0	5.6 ± 0.1	<0.001 ^2^
Average time of use on weekends (hours)	7.6	7.6 ± 0.1	7.6 ± 0.1	0.746 ^2^
Subjective health problems caused by smartphone use				
Not at all (%)	28,986 (55.0)	15,443 (54.7)	13,543 (55.3)	0.08 ^1^
No (%)	17,639 (33.3)	9495 (33.3)	8144 (33.3)	
Yes (%)	5279 (9.9)	2940 (10.2)	2339 (9.5)	
Always (%)	976 (1.9)	523 (1.8)	453 (1.9)	

The numbers of adolescents are presented as unweighted values and the percentages in parentheses as weighted values. The quantitative variables are calculated via complex-sample analysis and presented as the mean ± standard error of the mean. ^1^ Complex-sample chi-square test; ^2^ Complex-sample generalized linear model.

**Table 2:** Association between smartphone use and subjective health problem in 2023.

	Presence of Subjective Health Problems Caused by Smartphone Use in 2023 (*n* = 52,880)		
	**Odds Ratio**	**95% Confidence Interval**	***p* Value** ^1^
Educational stage (middle school)	1.110	1.041–1.184	<0.001
Smartphone overdependence (Presence)	9.940	9.299–10.625	<0.001
Average time of smartphone use on weekdays (h)	1.013	0.998–1.027	0.088
Average time of smartphone use on weekends (h)	1.038	1.027–1.050	<0.001

^1^ Complex-samples logistic regression analysis.

## Data Availability

All relevant data are included in the paper. Otherwise, the raw data analyzed during the current study are not publicly available as the owners provided their written consent only to the use of the data for the current study on IRB approval and for ethical reason. The datasets used and/or analyzed in the present study are available from the corresponding author on reasonable request.
